# Transcriptomic Characterization of the Porcine Urinary Bladder Trigone Following Intravesical Administration of Resiniferatoxin: Insights from High-Throughput Sequencing

**DOI:** 10.3390/toxins17030127

**Published:** 2025-03-09

**Authors:** Ewa Lepiarczyk, Mateusz Maździarz, Łukasz Paukszto, Agnieszka Bossowska, Mariusz Majewski, Jerzy Kaleczyc, Elżbieta Łopieńska-Biernat, Łukasz Jaśkiewicz, Agnieszka Skowrońska, Mariusz T. Skowroński, Marta Majewska

**Affiliations:** 1 Department of Human Physiology and Pathophysiology, School of Medicine, Collegium Medicum, University of Warmia and Mazury in Olsztyn, 10-082 Olsztyn, Poland; agnieszka.bossowska@uwm.edu.pl (A.B.); mariuszm@uwm.edu.pl (M.M.); lukasz.jaskiewicz@uwm.edu.pl (Ł.J.); agnieszka.skowronska@uwm.edu.pl (A.S.); marta.majewska@uwm.edu.pl (M.M.); 2 Department of Botany and Nature Protection, Faculty of Biology and Biotechnology, University of Warmia and Mazury in Olsztyn, 10-719 Olsztyn, Poland; mazdziarzm@gmail.com (M.M.); pauk24@gmail.com (Ł.P.); 3 Department of Animal Anatomy, Faculty of Veterinary Medicine, University of Warmia and Mazury in Olsztyn, 10-719 Olsztyn, Poland; jerzyk@uwm.edu.pl; 4 Department of Biochemistry, Faculty of Biology and Biotechnology, University of Warmia and Mazury in Olsztyn, 10-719 Olsztyn, Poland; ela.lopienska@uwm.edu.pl; 5 Department of Basic and Preclinical Sciences, Institute for Veterinary Medicine, Faculty of Biological and Veterinary Sciences, Nicolaus Copernicus University, 87-100 Torun, Poland; skowron@umk.pl

**Keywords:** resiniferatoxin, urinary bladder trigone, animal model, RNA-seq

## Abstract

Resiniferatoxin (RTX), a potent capsaicin analog, is being investigated as a therapeutic agent for neurogenic conditions, particularly those affecting bladder control. However, the transcriptomic effects of RTX on the urinary bladder remain largely unexplored. This study aimed to characterize the transcriptomic changes in the porcine urinary bladder trigone region removed seven days post-treatment with intravesical RTX administration (500 nmol per animal in 60 mL of 5% aqueous solution of ethyl alcohol). High-throughput sequencing identified 126 differentially expressed genes (DEGs; 66 downregulated, 60 upregulated), 5 differentially expressed long non-coding RNAs (DELs), and 22 other RNAs, collectively involved in 175 gene ontology (GO) processes. Additionally, differential alternative splicing events (DASes) and single nucleotide variants (SNVs) were detected. RTX significantly modulated signaling pathways related to nerve growth and myelination. Changes in genes associated with synaptic plasticity and neuromodulation were observed, particularly within serotoninergic and cholinergic signaling. RTX altered the expression of immune-related genes, particularly those involved in chemokine signaling and immune regulation. Notably, altered gene expression patterns suggest a potential anti-cancer role for RTX. These findings provide new insights into RTX’s therapeutic effects beyond TRPV1 receptor interactions, filling a critical gap in our understanding of its molecular impact on bladder tissue.

## 1. Introduction

Every year, millions of men and women worldwide suffer from dysfunctions of bladder control [1]. Proper urine storage and elimination, mediated by multiple neurotransmitters, is an intricate process, highly reliant on the sophisticated and coordinated activity of neurons distributed at many levels of the nervous system (including the brain, spinal cord, and peripheral nerves) [2]. These mechanisms require the undisturbed cooperation of autonomic, somatic, and afferent nerves regulating the micturition process. Norepinephrine (NE), released from the postganglionic sympathetic nerve fibers in the bladder body, mediates smooth muscle relaxation via the stimulation of the β-adrenergic receptors, while, on the contrary, causing contraction at the bladder base through binding to α-adrenergic receptors. Thus, sympathetic stimulation is mostly engaged in the urine storage mechanism. The parasympathetic transmission mediates bladder emptying, as the release of acetylcholine (ACh) from cholinergic neurons activates M2 and M3 muscarinic receptors and leads to contraction of the detrusor muscle [3]. Anticholinergic drugs are the primary treatment option for lower urinary tract symptoms (LUTS) characterized by frequent and sudden urges to urinate, such as those experienced by patients with overactive bladder (OAB). This effect is attributed to their ability to block the ACh action [4]. Unfortunately, their use does not always result in satisfactory improvement and is often limited by bothersome side effects such as dry mouth, blurred vision, cognitive changes, constipation, or urinary retention arising from the extensive blockade of cholinergic activity [5]. Moreover, most recent data dealing with LUTS pathomechanism suggest that uncoordinated afferent neurotransmission is the key factor underlying an uncontrolled and exaggerated micturition reflex [6]. This implies a need to develop effective drugs for urological disorders affecting sensory transmission. One such substance is resiniferatoxin (RTX), extracted from several plants belonging to the genus Euphorbia. RTX primarily targets transient receptor potential vanilloid type 1 (TRPV1) calcium channels on primary sensory neurons involved in nociception [7,8]. It is worth mentioning that RTX is a powerful analog of capsaicin. RTX elicits responses similar to those of capsaicin; however, RTX and capsaicin demonstrate differing relative potencies across a range of responses. Similarly to capsaicin, RTX treatment, after initial neuronal excitation triggered by channel opening and calcium influx, causes the defunctionalization of primary sensory fibers. The prolonged opening of calcium channels by RTX may lead to cytotoxicity in TRPV1-positive pain-conveying fibers, particularly after extended exposure or application to sensory neuron perikarya, ultimately resulting in cell death [7,9,10]. Although capsaicin was initially investigated for treating bladder disorders, RTX offers key advantages: it induces desensitization without pain and has a greater desensitization potency [11,12]. The main rationale behind using RTX in urology is its high selectivity towards TRPV1-containing peripheral nerve endings while retaining all other sensory modalities and motor function of the bladder [10], and current clinical studies support the effectiveness of this agent in the therapy of many disorders associated with LUTS [13,14].

The urinary bladder consists of the dome (also known as the body) located above the urethral orifices, and the base comprising the trigone and bladder neck [3]. In our recent study [15], we investigated the influence of the intravesical administration of RTX on the porcine urinary bladder wall samples (taken from the dome region). However, it is well documented, that the bladder base and trigone region in both animals and humans significantly differ in terms of their development, structure, innervation, and thus the role they play in the bladder filling and micturition process [16,17]. Unlike the bladder dome, the urinary bladder trigone (UBT), a region between two ureteral orifices and the internal urethral meatus, hardly stretches during the filling phase. However, it does play a supportive role in micturition and anti-reflux mechanism that prevents back-flow of urine to the ureters [16]. UBT is characterized by higher connective tissue content and higher spontaneous activity of muscle cells compared to dome [18]. Most probably, the contraction of UBT muscles facilitates bladder filling by opening the ureters and closing the bladder outlet [17]. Moreover, UBT is occupied by small diameter unmyelinated C-fibers which play a significant role in the pathomechanism of several bladder conditions including OAB [19] and interstitial cystitis/bladder pain syndrome [20]. These fibers are physiologically silent (they do not send signals) and do not take part in the micturition. However, they can become hyperactive under pathological conditions, contributing to urinary overactivity and increased pain sensation [21]. Such regional distribution of innervation allows for the application of anatomically specific therapeutic interventions in certain instances [20].

Although RTX is a promising therapeutic agent for lower urinary tract dysfunctions, its molecular effects on the UBT remains largely unexplored. A comprehensive understanding of RTX’s therapeutic effects on the UBT necessitates a thorough investigation of its molecular mechanisms. Our study addresses this gap by performing genome-wide deep RNA sequencing to identify transcriptomic changes induced by RTX in the porcine UBT. The study was performed on pigs, widely used in translational biomedical studies, as they, to some extent, resemble humans in terms of their anatomy, physiology, and genetics. This makes them one of the best animal models for use in biomedical research [22,23]. Specifically, the structure of the porcine urinary tract closely resembles that of humans, making them an excellent model for studying bladder function and intravesical drug administration. In addition, the relevant size of the animal enables the implementation of the intravesical route of RTX administration applied in humans, providing more relevant insights into therapeutic effects [24,25].

## 2. Results

### 2.1. Transcriptomic Signatures of Differentially Expressed Genes (DEGs) and Functional Annotations

**Figure 1:**
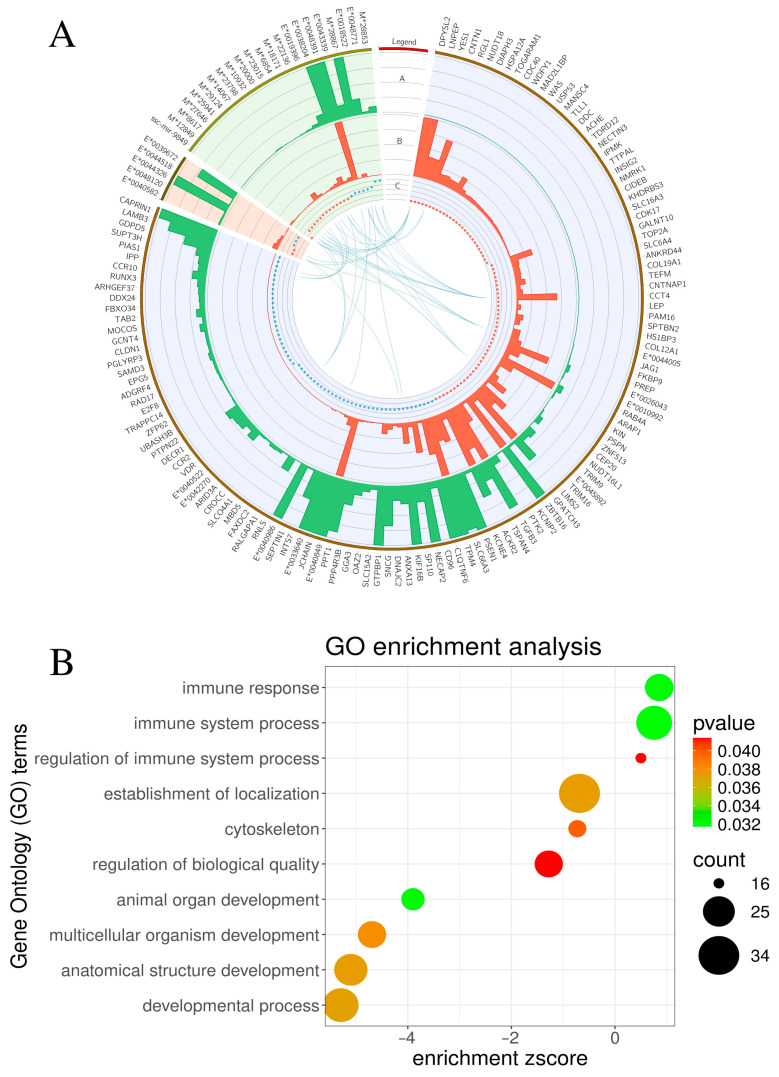
(**A**) Circos plot: track A presents bar plots of the mean expression from resiniferatoxin (RTX)-treated urinary bladder trigones, while track B presents the mean expression from control (CTR) samples. Track C shows a log2FoldChange scatter plot, with blue dots representing values > 1 and red dots representing values < −1. The innermost track represents links that reflect the correlation between differentially expressed genes (DEGs)—differentially expressed long non-coding RNAs; (DELs) (green); and DEGs—other RNA (blue). (**B**) The dot plot presents the 10 statistically significant processes with the highest contribution of statistically significant genes. The size of the dot represents the number of genes in the process, while the color of the dot represents the *p*-value, with smaller *p*-values being greener and larger *p*-values being redder. Notably, RTX altered the expression of immune-related genes, particularly those involved in immune response and regulation of the immune process. Process names are displayed on the left side of the plot, and the *x*-axis represents the enrichment z-score.

The summary statistics of 12 cDNA libraries (six from RTX-UBT and six from controls; CTR-UBT) are described in Table S1. Following high-throughput sequencing, 749,768,700 paired-end reads were processed, yielding 623,139,874 clean reads. We mapped more than 80.55% of the reads to the Sus_Scrofa.11.1.104 genome, with mapping findings indicating over 96.65% of uniquely matched sequences. RNA-seq revealed data on 153 molecules with statistically significant expression variations (Table S2). A total of 126 differentially expressed genes (DEGs), 5 differentially expressed long non-coding RNAs (DELs), and 22 other RNAs were identified among these transcripts. We discovered 66 downregulated and 60 upregulated genes among the DEGs. Conversely, there were two downregulated and three upregulated DELs. The additional RNA collection comprised 14 downregulated and 8 upregulated components. All of these differentially expressed genes participated in 175 gene ontology (GO) processes, comprising 56 biological processes, 94 cellular components, and 25 molecular functions. The ‘immune system process’ contained 28 major molecules, the ‘immune response’ included 22 significant molecules, and the ‘regulation of immune system’ process comprised 16 significant molecules (Table S3). Moreover, the genes participated in other notable processes, including ‘anatomical structure development’, ‘animal organ development’, ‘cytoskeleton’, ‘developmental process’, ‘establishment of localization’, ‘multicellular organism development’, and ‘regulation of biological quality’, which exhibited the highest quantity of statistically significant molecules involved. We discovered four statistically significant associations between DELs and DEGs: ENSSSCG00000040582 and DPYSL2 (r Pearson = 0.85), ENSSSCG00000040582 and FKBP9 (0.81), ENSSSCG00000040582 and ANXA13 (0.83), and ENSSSCG00000048120 and DPYSL2 (0.90). Furthermore, 34 associations were identified between DEGs and other RNA molecules. The genes DPYSL2 and CCT4 exhibited the highest number (7) of linkages with other RNA based on expression profiles (Table S4). The ggplot2 version 3.5.1 software visually depicted the outcomes of the functional analysis (Figure 1).

### 2.2. Alternative Splicing

**Figure 2:**
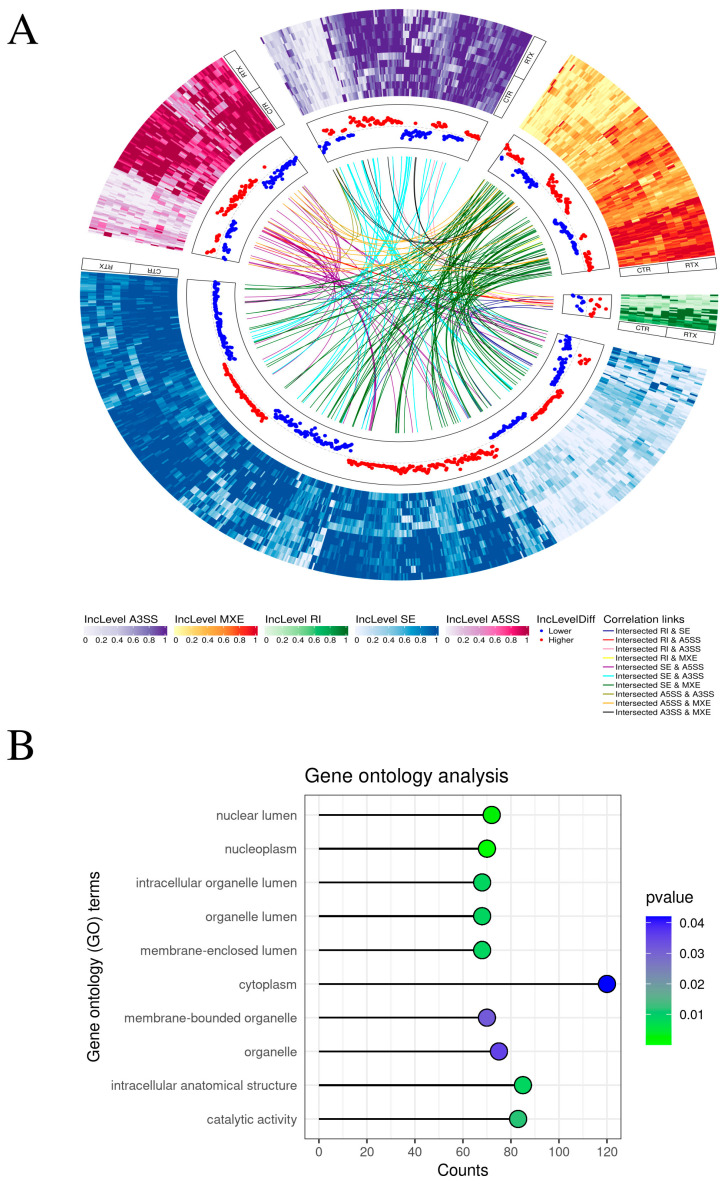
(**A**) Circular visualization representing all statistically significant differential alternative splicing events (DASes) between the resiniferatoxin (RTX)-treated urinary bladder trigones and control samples (CTR). The outermost track is a heatmap that visualizes the inclusion of each event, and the color of each heatmap corresponds to the type of splicing event: green for retained intron (RI) events, blue for skipping exon (SE) events, pink for alternative 5′ splice sites (A5SS), purple for alternative 3′ splice sites (A3SS), and orange for mutually exclusive exons (MXE). The next track shows a dot plot representing the IncLevelDifference. If it is lower than 0.1, the dot is blue; if it is higher than 0.1, the dot is red. The innermost track shows common splicing events for the same gene. (**B**) The dot plot presents the 10 statistically significant processes with the highest contribution of statistically significant DASes. The color of the dot represents the *p*-value, with smaller *p*-values being greener and larger *p*-values being bluer. Process names are displayed on the left side of the plot, and the *x*-axis represents the number of genes in the process. Most enriched GO terms included: ‘Cytoplasm’, ‘Intracellular anatomical structure’ and ‘Catalytic activity’.

An extensive examination of alternative splicing events (ASEs) was performed, uncovering 1058 splicing events distributed among several categories: 167 alternative 3′ splice sites (A3SS), 144 ASEs as 5′ splice sites (A5SS), 163 mutually exclusive exons (MXE) events, 24 retained intron (RI) events, and 560 skipping exon (SE) events (Figure 2A and Table S5). For 536 events, IncLevelDiff was larger than 0.1, while for 522 events, it was less than −0.1. All splicing events associated with gene symbols were implicated in 76 statistically relevant GO processes. This comprised 3 biological processes (BP), 34 cellular components (CC), and 39 molecular functions (MF) (Figure 2B and Table S6). The GO terms with the greatest number of linked events were ‘cytoplasm’, ‘intracellular anatomical structure’, and ‘catalytic activity’.

### 2.3. Differential Single Nucleotide Variants

**Figure 3:**
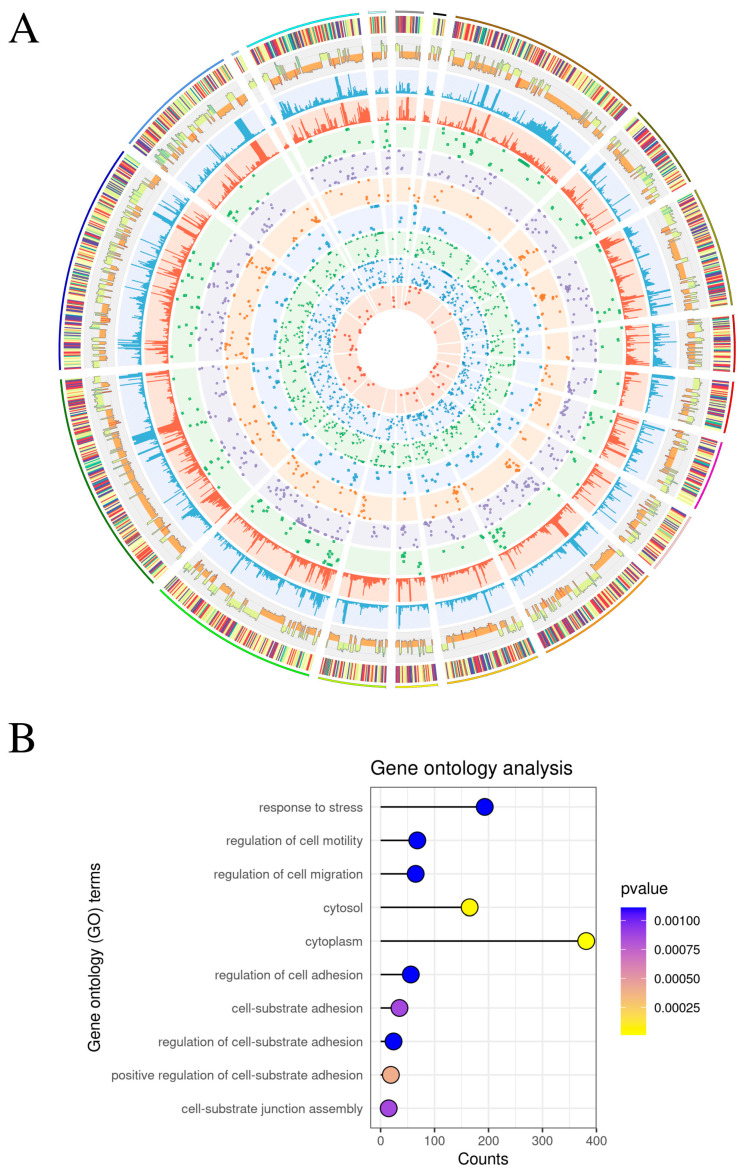
(**A**) The circular plot displays highly significant allele-specific expression events (FDR < 0.0001, absolute value of alternative allele fraction difference > 0.3) across 12 trials, including 6 resiniferatoxin (RTX)-treated urinary bladder trigones and 6 control (CTR) samples. The colors of the first heatmap represent substitution fractions. The second path highlights the difference in alternative allele fraction difference between the higher and lower allele-specific expression events in each compared group. The next two tracks represent coverage, with blue representing the reference allele and red representing the alternative allele. The last six internal paths demonstrate the type of single nucleotide variant (SNV). (**B**) The dot plot presents the 10 statistically significant processes with the highest contribution of significant SNVs. The color of the dot represents the *p*-value, with smaller *p*-values being yellower and larger *p*-values being bluer. Process names are displayed on the left side of the plot, and the *x*-axis represents the number of genes in the process. In total, 483 gene ontology (GO) processes were enriched, including biological processes (‘stress response’, ‘apoptosis’, ‘neuron development’, ‘axonogenesis’, and ‘immune regulation’), cellular components (‘cytoplasm’ and ‘endoplasmic reticulum’), and molecular functions (‘acetyltransferase activity’).

The variant calling process detected 1778 polymorphisms, underscoring the possibility for RNA heterogeneity and likely particular expression bias subsequent to RTX treatment. Variable single nucleotide variants (SNVs) demonstrated that their 5372 annotations influenced gene sequence regions. High-scoring single nucleotide variations linked to RTX care were identified in 950 unigenes. In the RTX group, elevated alternative allele frequencies in 1052 SNVs were considered significant in the expressed transcripts. Conversely, 726 polymorphic sites exhibited distinct expression within the CTR group. The examination of the identified heterozygous RNA locations uncovered 1334 synonymous variants, 473 missense variants, 1523 3′ UTR variants, and 104 5′ UTR variants (Figure 3A and Table S7). Functional categorization categorized differential SNVs into 483 statistically relevant GO processes. The GO BP category was enriched in 425 processes, including: ‘response to stress’ (GO:0006950; 193 allele-specific variants), ‘apoptotic process’ (GO:0006915; 85 allele-specific variants), ‘regulation of programmed cell death’ (GO:0043067; 68 allele-specific variants), ‘regulation of neuron projection development’ (GO:0010975; 17 allele-specific variants), ‘regulation of axonogenesis’ (GO:0050770; 13 allele-specific variants), ‘regulation of leukocyte chemotaxis’ (GO:0002688; 11 allele-specific variants), ‘axon extension’ (GO:0048675; 7 allele-specific variants), ‘negative regulation of axonogenesis’ (GO:0050771; 8 allele-specific variants), ‘preganglionic parasympathetic fiber development’ (GO:0021783; 4 allele-specific variants), ‘axonal fasciculation’ (GO:0007413; 3 allele-specific variants), and ‘neuron projection fasciculation’ (GO:0106030; 3 allele-specific variants). The GO CC category was enriched with 41 terms, including ‘cytoplasm’ (GO:0005737; 120 allele-specific variations) and ‘endoplasmic reticulum’ (GO:0005783; 80 allele-specific variants). The GO MF category was enriched in 17 processes, including ‘acetyltransferase activity’ (GO:0016407; 7 allele-specific variations) (Figure 3B and Table S8).

### 2.4. Multi-Level Transcriptomic Modification

**Figure 4:**
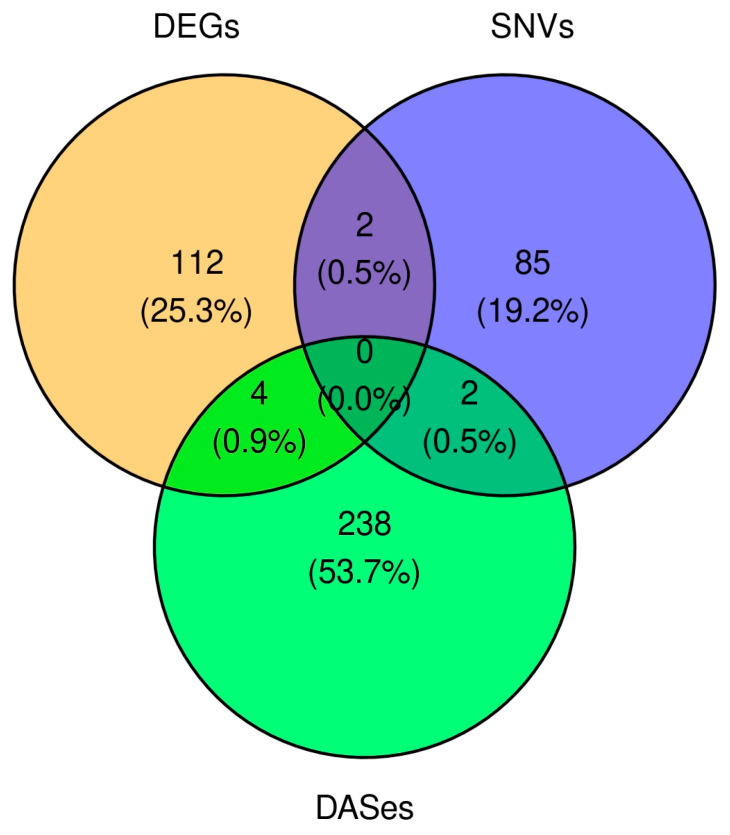
Venn diagram illustrating the comparison of all analyses. The comparison was performed at the gene symbol level from the ENSEMBL database. The green color on the Venn diagram represents differential alternative splicing events (DASes), blue represents single nucleotide variants (SNVs), and orange represents differentially expressed genes (DEGs). An analysis of all omics data identified four genes that were both DEGs and DASes. DASes and SNVs were discovered in the two genes. Alterations in DEGs and SNVs were observed in two genes.

An analysis of all omics data identified four genes that were both DEGs and alternative splicing events (DASes). The DEGs group comprised ACKR2, which exhibited a statistically significant splicing event (RI) with an IncLevelDiff of 0.11 and a downregulated log2FoldChange of −1.21. The remaining three genes were: KIF16B (SE, IncLevelDiff = −0.18, log2FoldChange = 1.97), DNAJC2 (A3SS, IncLevelDiff = 0.147, log2FoldChange = 2.72), and TAB2 (MXE, IncLevelDiff = 0.144, log2FoldChange = 23.6). DASes and SNVs were also discovered in the genes MIF and CAMK2D. Alterations in DEGs and SNVs were also observed in LNPEP and WDFY1 (Figure 4).

### 2.5. Validation of the Results

**Figure 5:**
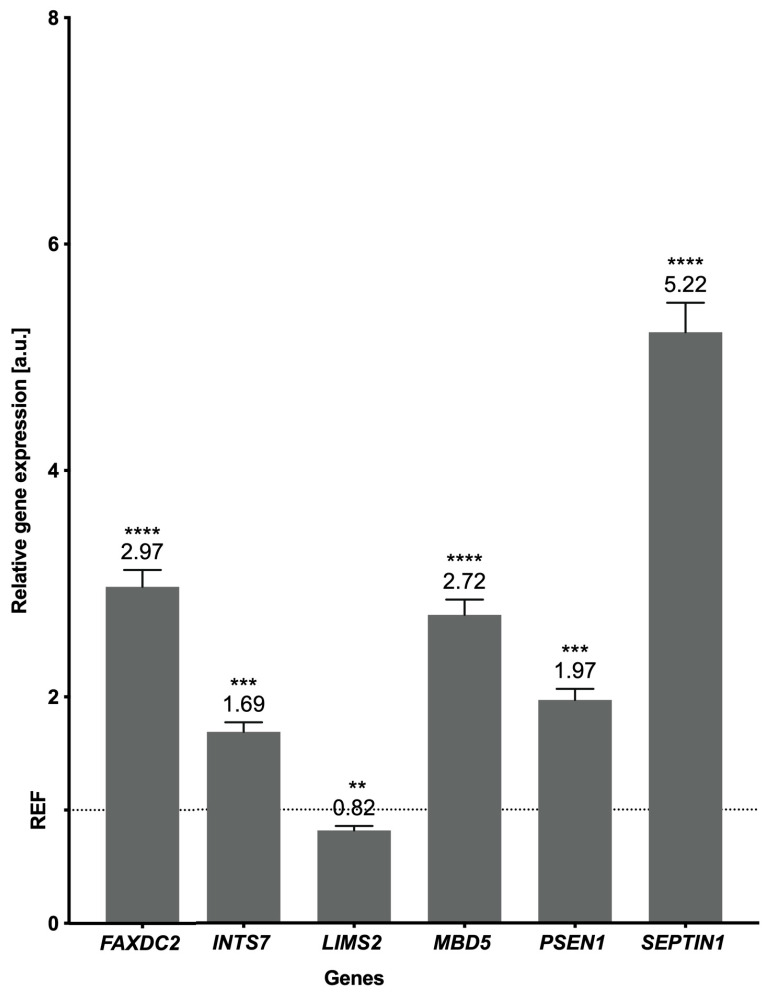
The mRNA expression of selected genes obtained using real-time PCR. The expression of endogenous control is shown as normalized to a value 1 (REF/dashed line), and the expression of genes of interest indicates the changes relative to the control. The exact values of expression are visible above the bars. *p*-values ≤ 0.05 were considered statistically significant where ≤0.0021 (**), ≤0.0002 (***), and ≤0.0001 (****).

The NGS approach was confirmed using real-time PCR, which demonstrated the overexpression of five genes—FAXDC2, INTS7, MBD5, PSEN1, and SEPTIN1—and the underexpression of LIMS2 (Figure 5). Genes were validated according to their functionality, expression levels, and distribution across samples. MBD5 and SEPTIN1, which are overexpressed, have been identified as crucial factors in neurogenesis and the control of synaptic activity. SEPTIN1 and FAXDC2 [26,27] have been previously associated with immune response pathways, whereas FAXDC2, INTS7, PSEN1, and LIMS2 influence the carcinogenic process [28,29,30,31].

### 2.6. The Distribution and Relative Frequency of 5-HT-Immunoreactive Nerve Fibers

**Figure 6:**
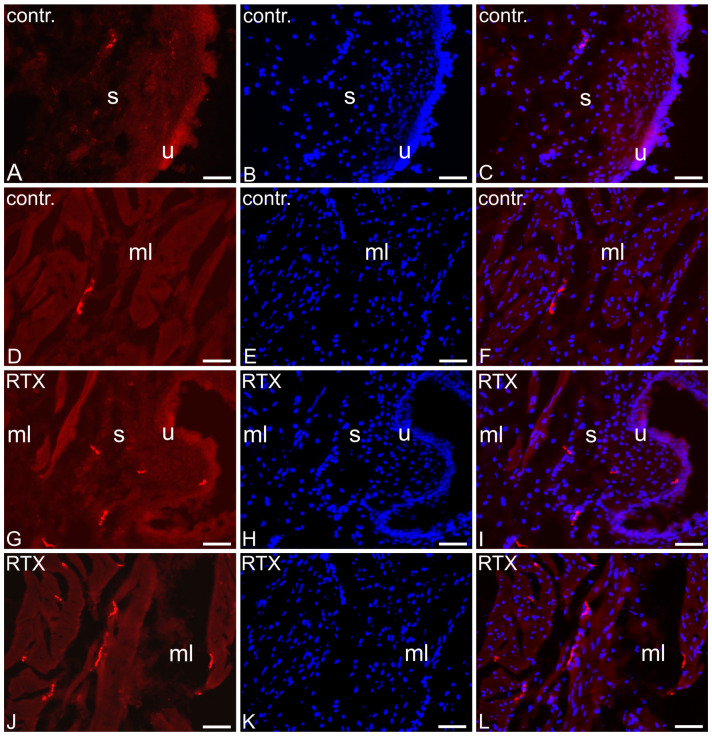
Columns from the left. First column: representative images of the distribution and relative frequency of serotonin (5-HT)—immunopositive nerve terminals in the control (contr.; (**A**,**D**)) and resiniferatoxin-treated (RTX; (**G**,**J**)) urinary bladder trigones (images taken separately from red fluorescent channel). Second column: nuclei stained with DAPI, visualized as blue fluorescence ((**B**,**E**,**H**,**K**); images taken separately from blue fluorescent channel). Third column—merged images of red and blue fluorescent channels (**C**,**F**,**I**,**L**). Muscle layer (ml), submucosa (s), urothelium (u); 20×; Bar in all images—50 µm.

In the control pigs, only single 5-HT-immunoreactive NF were observed in all the layers of the UBT (muscle layer, submucosa, and urothelium; Table 1; Figure 6A–C).

After RTX intravesical treatment, the number of 5-HT-immunopositive NF in the smooth muscle layer was higher than that observed in the CTR pigs (a moderate number of these nerve terminals were found in this layer). Also, a slightly higher number of 5-HT immunopositive nerve terminals was observed after RTX treatment in the submucosa and penetrating the urothelium (Table 1; Figure 6D–F).

## 3. Discussion

### 3.1. Transcriptomic Changes Following RTX Administration Suggest Altered Expression of Genes Related to the Affected Sensory Fibers and Nerve Degeneration Onset

The primary justification for employing RTX in urology is its selective targeting of TRPV1-positive peripheral nerve endings, thereby avoiding interference with other sensory modalities or bladder motor function [13]. TRPV1 exhibits widespread tissue distribution. As far as the urinary bladder is concerned, this channel is expressed at the highest levels in a subpopulation of primary afferent sensory neurons projecting to this organ; however, it is also present in nonneural structures, such as urothelial and interstitial cells [32]. The main action of RTX concerning TRPV1 involves the prolonged channel opening, resulting in a significant rise in the intracellular calcium level which is cytotoxic to primary sensory neurons [33]. Topical application of RTX has been shown to induce defunctionalization and potential axonal degeneration at the site of application, particularly in the bladder mucosa and muscular layer [9]. These changes undoubtedly lead to altered expression of mRNAs in the affected sensory nerve cells and their projections. Studies have demonstrated that intravesical administration of RTX leads to a significant reduction in calcitonin gene-related peptide (CGRP) and substance P (SP) immunoreactive nerve fibers in both the mucosa and muscular layers of the bladder. This reduction suggests defunctionalization of sensory afferent fibers. Notably, while the number of these fibers decreased to less than 20% of control levels within 24 h, they returned to normal levels by the eighth week [34]. Further research involving intravesical RTX in healthy cats revealed a decrease in SP and CGRP nerve axon density in the bladder tissue. Moreover, mild to moderate histopathological changes, including epithelial alterations, edema, and blood vessel proliferation, were noted, with more pronounced effects at higher doses. These findings, once again, indicate that RTX can induce desensitization of sensory nerves and axonal degeneration at the application site [35]. RTX administration can also have indirect effects on various cell types, including Schwann cells, urothelial cells, and smooth muscle cells. Although Schwann cells do not express TRPV1 receptors, RTX-induced activation of sensory neurons can lead to the release of neuropeptides and other signaling molecules, which may influence their function and nerve regeneration processes. Studies have shown that urothelial cells also express TRPV1 receptors, suggesting that RTX could directly affect their function. Activation of TRPV1 in these cells may modulate bladder reflexes and influence conditions such as overactive bladder syndrome [36]. Smooth muscle cells do not typically express TRPV1 receptors. However, RTX-induced activation of sensory neurons can result in the release of substances like CGRP and SP, which can cause vasodilation and influence smooth muscle contractility indirectly [37].

In our previous study [15], where we examined the molecular influence of RTX on the urinary bladder dome, we identified several DEGs associated with neurodegenerative pathways. Nevertheless, some detected DEGs were found to be linked to the initiation of neuroprotective responses, indicating the potential for compensatory mechanisms. Consequently, the findings of this study, focusing on the impact of RTX on the UBT, have demonstrated that similar changes occur in response to the toxin. Thus, in our opinion, the present data supports the thesis that the transcriptomic changes observed in the present study predominantly involve changes resulting from the altered expression of genes related to the affected sensory fibers. A manifestation of the onset of nerve degeneration after the toxin administration can be seen in modifications in several genes associated with nerve growth or apoptosis processes. One such example is the downregulated Persephin (*PSPN*), likely suggesting neuronal damage caused by the toxin, given its role as a gene encoding a neurotrophic factor with neuroprotective and neurodegenerative properties [38]. In the RTX-treated UBT, not only underexpression but also the presence of SNV pertained to WD repeat and FYVE domain containing 1 (*WDFY1*), the downregulation of which has been previously found to inhibit neurogenesis [39]. It should be noted that the functional annotation classified this gene into the ‘response to stress’ and ‘regulation of response to stress’ categories. Moreover, DASe and SNV were identified in macrophage migration inhibitory factor (MIF), a gene assigned to the ‘regulation of programmed cell death’, enriched in the RTX-treated samples.

In the RTX-treated UBT, the reflection of the activation of cellular pathways underlying neuroregeneration following apoptotic cell death is indicated, for instance, by the upregulation of methyl-CpG binding domain protein 5 (*MBD5*), a protein-coding gene, which functions in transcriptional regulation. Disruptions in *MBD5* have been associated with various neurodevelopmental disorders [40] and MBPs, such as MBD5, are essential for DNA methylation, a critical process in neurogenesis [41]. Additionally, RTX installation led to a decrease in the expression of Dihydropyrimidinase Like 2 (*DPYSL2*), a gene implicated in apoptosis. Remarkably, our study identified four statistically significant correlations between DELs and DEGs, including *DPYSL2* and *ENSSSCG00000040582* and *DPYSL2* and *ENSSSCG00000048120*.

### 3.2. Potential Anti-Cancer Mechanisms of Intravesical RTX Administration

Interestingly, *DPYSL2* was also classified as one of 12 genes significantly associated with the prognosis of bladder cancer patients, and its high expression is associated with a favorable prognosis in bladder cancer patients [42]. It has been revealed that *DPYSL2* was significantly upregulated in urinary bladder cancer compared to normal tissue, and the higher the expression, the more advanced the cancer stage. Moreover, *DPYSL2* deficiency inhibited the malignant behavior and epithelial–mesenchymal transition in bladder cancer cells [43], thus the downregulation of *DPYSL2* observed in the UBT after RTX administration may suggest the anti-cancer properties of the toxin. In urology, RTX is mostly used for the treatment of functional neurologic dysfunctions. Cancer studies investigating RTX primarily explore its analgesic properties for managing severe pain in advanced stages [44]. However, a recent study by Farfariello et al. [45] has also explored the potential anti-cancer effects of RTX, particularly in bladder cancer. This study demonstrated that RTX induces cell death in bladder cancer cells through mitochondrial dysfunction, leading to increased reactive oxygen species production and altered ADP/ATP ratios. In vivo, RTX administration resulted in reduced tumor growth in a xenograft mouse model of bladder cancer. These findings suggest that RTX’s cytotoxic effects on bladder cancer cells are mediated by disrupting mitochondrial function. While these studies provide preliminary evidence supporting RTX’s anti-cancer potential in bladder cancer, further research is necessary to fully elucidate its therapeutic efficacy and safety in clinical settings. The present study has also identified genes other than *DPYSL2*, whose altered expression may reflect RTX anti-cancer properties. For instance, RTX administration has been followed by the downregulation of the chaperonin containing TCP1 subunit 4 (*CCT4*) gene. Interestingly, the current research revealed 34 correlations between DEGs and other RNA in RTX UBT, and *DPYSL2* and *CCT4* genes formed the most connections based on expression profiles with other RNA. CCT4 is a part of the chaperonin-containing t-complex significant in cell cycle-related protein degradation [46]. *CCT4* overexpression has been revealed in cancer cells compared with normal tissue, and the lack of *CCT4* was found to promote apoptosis of cancer cells [47]. Thus, the observed downregulation of the CCT4 is consistent with RTX’s potential anti-cancer role. Moreover, the current data revealed the downregulation of DNA topoisomerase II Alpha (*TOP2A*) in RTX-treated UBT. In urinary bladder cancer, TOP2A has been suggested to be one of the potential therapeutic targets and a prognostic biomarker, as its overexpression has been strongly linked to the progression of the tumor stage and poor prognosis [48]. Moreover, in bladder urothelial carcinoma, the knockdown of *TOP2A* significantly reduced cancer cell proliferation [49]. The overexpression of this gene has been reported in several cancer types, including bladder cancer, and its upregulation has related to an increased risk of metastasis due to intensified migration of cells and inhibited apoptosis [48]. Additionally, the current study revealed other DEGs previously linked with cancer, such as the downregulated genes LIM zinc finger domain containing 2 (*LIMS2*; also known as *PINCH2*) and jagged canonical Notch ligand 1 (*JAG1*). It has been found that LIMS2 is involved in cell migration and adhesion via integrins and its deletion or downregulation correlated with promoting invasion and metastasis in several cancer types [31,50]. On the other hand, JAG1 is a critical Notch ligand that initiates Notch signaling through cell–cell interactions. Its overexpression has been documented across multiple cancer subtypes and is correlated with a poor clinical prognosis. Additionally, JAG1/Notch signaling pathways activate multiple oncogenic factors that govern cellular processes including proliferation, metastasis, chemoresistance, and angiogenesis. Consequently, the reduced expression of *JAG1* may potentially suppress JAG1/Notch signaling activity and exhibit an anti-cancer effect. In the RTX-treated UBT, the current study revealed the upregulation of presenilin 1 (*PSEN1*). Presenilins are essential functional components of γ-secretase, an enzyme responsible for cleaving several transmembrane proteins. Studies have implicated PSEN1 dysfunction in both Alzheimer’s disease and cancer [51,52,53]. Growing evidence suggests a role for presenilins in carcinogenesis; however, their precise function in cancer development remains unclear. For instance, it has been found that PSEN1 overexpression reverses multi-chemoresistance in breast cancer by DNA initiating the damage response pathway [54]; conversely, PSEN1 expression was notably elevated in patients diagnosed with gastric cancer [53].

### 3.3. An Immunomodulating Effect Exerted by RTX in the Treated UBT

#### 3.3.1. TRPV1 and Its Role in Immune System Modulation

TRPV1, while primarily recognized for its role in pain and thermal sensation, has been demonstrated to exhibit a multifaceted functional profile, encompassing additional critical cellular processes such as proliferation and apoptosis. Furthermore, TRPV1 has been shown to participate in the modulation of immune response [55]. Studies have shown that this channel’s expression is elevated in various chronic inflammatory diseases, suggesting a pro-inflammatory role [56]. However, recent findings also point to its anti-inflammatory and protective functions [57]. TRPV1 is involved in the inflammation process at several various levels, including modulations of regulatory proteins and inflammatory mediators of the immune response, and the outcome of its response depends on internal and external factors influencing its activity [55].

#### 3.3.2. Immune-Related Gene Expression Changes Following RTX Treatment

The findings of this study provide evidence in support of the hypothesis that by acting on TRPV1 receptors, RTX significantly modulates the immune reaction in the treated tissues. In the UBT, the RTX intravesical administration was followed by differential expression of several genes strongly associated with immune response, and the GO functional analysis of the obtained DEGs revealed that they were engaged in GO BP including ‘immune system process’, ‘immune response’, ‘immune effector process’, ‘regulation of immune system process’, ‘regulation of immune response’, ‘regulation of immune effector process’. Interestingly, such a strong impact on the immune system was not observed in bladder wall fragments collected from the dome region [15].

#### 3.3.3. Chemokine Signaling and Immune Regulation

Several DEGs revealed in the RTX-treated UBT, such as the upregulated C-C motif chemokine Receptors 2 (*CCR2*) and 10 (*CCR10*), joining chain of multimeric IgA and IgM (*JCHAIN*), and downregulated atypical chemokine receptor 2 (*ACKR2*), encode proteins associated with the function of chemokines. The crucial role of chemokines in eliciting immune responses and regulating pain sensitivity is widely acknowledged. For instance, CCR2 is a chemokine receptor found in many subpopulations of sensory fibers, which after activation is capable of sensitization of nociceptors via activation of TRPV1 receptors [58]. This result may be surprising, taking into account the blocking action of RTX on TRPV1 channels. However, CCR2 upregulation has been observed in sensory neurons following their injury [59], and thus in the present study it most probably results from sensory nerve fiber degeneration due to the toxin action. Interestingly, *ACKR2* was not only downregulated following RTX treatment, but also alternatively spliced. This gene encodes an atypical chemokine receptor, which can bind to several various chemokines and thus carry numerous different functions in the organism.

#### 3.3.4. Other Key Immune-Related Genes Affected by RTX

In RTX-treated UBT, the administration of the toxin led to the altered expression of numerous other genes associated with the regulation of immunological processes, including the upregulation of septin 1 (*SEPTIN1*) and fatty acid hydroxylase domain containing 2 (*FAXDC2*). The best-known function of septins is in cytokinesis, and in humans SEPTIN1 is highly expressed in lymphoid tissue [26]. Additionally, septins play an important role in neuronal morphogenesis, including axonal and dendritic arborization and modulation of synaptic activity [60]. *FAXDC2* encodes a hydroxylase enzyme involved in the synthesis of cholesterol and sphingomyelin. The overexpression of FAXDC2 has been previously linked with immune response mechanisms, as it promotes the differentiation of megakaryocytic cell lines and thus megakaryocyte maturation [27]. In addition, the downregulation of this enzyme has been revealed in several different types of cancer, whereas overexpression has the potential to inhibit the proliferation and invasive capacity of cancer cells [28].

### 3.4. Differential Expression of Genes Involved in Synaptic Plasticity and Neuromodulation

Interestingly, several DEGs identified in the UBT after RTX treatment were associated with synaptic signaling. The current research revealed the downregulation of solute carrier family 6 member 4 (*SLC6A4*). This gene encodes an integral membrane protein responsible for transport of the serotonin (5-HT) from synaptic spaces into presynaptic neurons, thus terminating the action of serotonin [61]. Thus, on the contrary, the underexpression of *SLC6A4* may result in decreased reuptake of the neurotransmitter and its prolonged action. It is noteworthy that our earlier investigation concerning the influence of intravesical RTX administration on the samples of the urinary bladder wall taken from the dome region revealed four genes enriched in the ‘serotoninergic synapse’ KEGG pathway [15]. Thus, the results imply that RTX exerts its therapeutic effect by modulating/enhancing serotoninergic transmission both in the bladder wall and trigone. Our previous research examined the impact of RTX intravesical instillation on the number and chemical coding of noradrenergic and cholinergic nerve fibers innervating the UBT in female pigs [62]. It is worth mentioning that these data were in line with the earlier observation of Sann et al. [63] who revealed that noradrenergic innervation is affected following ablation of the sensory innervation of this organ by the TRPV1 agonist capsaicin. While our previous study did not examine 5-HT transmission, the current findings demonstrated a significant effect of the toxin treatment on this neurotransmitter. To address this, we conducted additional immunohistochemical staining to investigate 5-HT distribution. The obtained results indeed confirmed that after the toxin treatment, the number of 5-HT-immunoreactive nerve terminals was higher in all the layers of the urinary bladder trigone (smooth muscle, submucosa, and urothelium). It can be assumed that the increased number of these axons reflects enhanced serotonin production, which can have significant implications for therapeutic approaches, particularly in urinary bladder disorders. It should be noted that 5-HT, by activating its receptors, modulates the micturition reflex by influencing both detrusor muscle contractility and nociception. Nevertheless, it must be stressed that the impact of 5-HT on pain sensation and micturition process is receptor subtype-dependent. This complexity arises from the existence of 14 identified 5-HT receptor types, organized into seven families [64]. 5-HT has been found to promote micturition and bladder contraction across multiple species, including humans [65]. Recio et al. [66] have found that 5-HT mediates relaxation of the pig urinary bladder neck. What is more, the first drug employed in the treatment of incontinence was imipramine, a serotonin reuptake inhibitor exhibiting sympathomimetic and anticholinergic properties [67]. To date, the pharmacological agents capable of modifying serotonergic transmission are one of the therapeutic options to treat LUTS [68]. It can be therefore concluded that an increased number of serotonergic fibers observed after RTX treatment in the present study could lead to improved modulation of bladder activity, potentially alleviating symptoms associated with urinary bladder disorders. However, this promising therapeutic implication of RTX requires further studies.

Finally, the current research demonstrated that the intravesical administration of RTX resulted in a reduction in the Acetylcholinesterase (*ACHE*), which encodes the enzyme responsible for hydrolysis and subsequent control of the amount of ACh at the neuromuscular junction [69]. The result was somewhat surprising, considering our previous research into the distribution, frequency, and chemical coding of cholinergic NF in the porcine UBT after RTX treatment [51], in which the intravesical instillation of the toxin resulted in a reduction in cholinergic NF innervating the smooth muscle layer. Thus, possibly the decrease in *ACHE* observed in the current study mirrors the feedback mechanism activated in response to neurodegeneration and the subsequent decline in ACh production caused by the toxin treatment. Interestingly, the study also identified several DASes that were subjected to GO functional analysis. The findings of this analysis pointed to six DASes, namely O-linked N-acetylglucosamine (*GlcNAc*) transferase (*OGT*), TATA-box binding protein associated factor 5 (*TAF5*), Mbt domain containing 1 (*MBTD1*), MSL complex subunit 3 (*MSL3*), transcriptional adaptor 1 (*TADA1*), and MSL complex subunit 1 (*MSL1*), which were engaged in the ‘protein acetyltransferase complex’ that catalyzes the transfer of an acetyl group to a protein acceptor molecule. This result further emphasizes the significant role of RTX in modulating cholinergic transmission, with potential implications for clinical applications.

## 4. Conclusions

In conclusion, this study conducted a genome-wide deep RNA sequencing analysis of UBT from both CTR and RTX-treated animals, aiming to identify specific transcriptional alterations within these tissues. A comprehensive analysis of the identified transcripts revealed that RTX treatment significantly modulated signaling pathways associated with the process of nerve degeneration. However, a subset of these DEGs was also implicated in the initiation of neuroprotective mechanisms. Additionally, intravesical administration of RTX resulted in changes in the expression of genes involved in synaptic plasticity and neuromodulation, including those related to serotonergic and cholinergic pathways. Furthermore, the investigation demonstrated that RTX exerts an immunomodulating effect in the treated UBT. Finally, several transcripts identified in the RTX-treated UBT suggest potential anti-cancer mechanisms associated with the toxin treatment.

## 5. Materials and Methods

### 5.1. Laboratory Animals

Twelve juvenile (8–12 weeks old, 15–20 kg bodyweight, b.w.) Large White Polish breed female pigs (animal source: livestock farm Agnieszka Tokarska-Dziąba, Tuszewo 60, 14-260 Lubawa, Poland; PL 038073830001) were used in this study. The animals were maintained in a standard laboratory setting. They received a commercial feed (Grower Plus, Wipasz, Wadazg, Poland) and had unlimited access to water. Pigs were transported from the breeder to the animal quarters 5 days before the procedure for acclimatization. The pigs were randomly assigned to two treatment groups. One group of six pigs (CTR) was given the intravesical instillation of a 5% aqueous ethyl alcohol solution (60 mL). The remaining six pigs were administered RTX (product code: AG-CN2-0534, AdipoGen Life Sciences, Fuellinsdorf, Switzerland) by intravesical instillation of the toxin (500 nmol per animal in 60 mL of 5% aqueous solution of ethyl alcohol) to employ a dosing regimen and administration route that closely resembles human clinical practice. Alcohol and RTX solutions were both applied under anesthesia. Initially, all animals received atropine (Atropinum Sulfuricum, Polfa, Warsaw, Poland, 0.05 mg/kg b.w., s.c.) and azaperone (Stresnil, Janssen Pharmaceutica, Belgium, 2.5 mg/kg b.w., i.m.) pretreatment. Atropine is an anticholinergic agent that reduces salivation and bronchial secretion, while azaperone is a butyrophenone neuroleptic sedative extensively used in pigs to prevent fighting and stress [70]. Subsequently, buprenorphine (Bupaq, Richter Pharma AG, Austria, 20 µg/kg b.w., m.c., i.m.) was administered to alleviate visceral pain. Anesthesia and analgesia were initiated 30 min later with intramuscular injection of ketamine (Bioketan, Vetoquinol, Poland, 10 mg/kg b.w.), xylazine (VetaXyl, Vet Agro, Poland, 0.15 mg/kg b.w.), and by propofol (Propofol-Lipuro, B. Braun Melsungen AG, 10 mg/kg b.w, Maria Enzersdorf, Austria) given intravenously in a slow, fractionated infusion. Anesthesia depth was assessed via corneal reflex testing. Seven days post-treatment with an aqueous solution of ethyl alcohol or RTX, a midline laparotomy was performed (all the animals received the same premedication and anesthesia as in the previous procedure). In each pig, the urinary bladder was carefully accessed and the UBTs (located between two ureteral orifices and the internal urethral meatus) were cut out to perform transcriptome sequencing. In vivo sampling was performed to obtain high-quality, pure RNA for sequencing. Subsequently, euthanasia was performed on all pigs via an overdose of sodium pentobarbital (Euthasol, FATRO, Ozzano dell’Emilia BO, Italy, 140 mg/kg). All the experimental protocols and methods were conducted following the Declaration of Helsinki and approved by the Ethics Committee for Animal Experimentation in Olsztyn, Poland (affiliated with the National Ethics Committee for Animal Experimentation, Polish Ministry of Science and Higher Education; decision No. 39/2020 from 22 July 2020). All methods were reported under ARRIVE guidelines.

### 5.2. RNA Extraction, Library Construction, and Sequencing

Among all the RTX-treated and CTR pigs (*n* = 12) total RNA was isolated from the collected UBTs. Total RNA was extracted using a mirVana kit following the manufacturer’s procedure (Thermo Fisher Scientific, Waltham, MA, USA). The total RNA isolates’ quantity and quality were evaluated using the Bioanalyzer 2100 (Agilent Technologies, Santa Clara, CA, USA). The samples with the highest RIN values and concentrations were selected for RNA-seq library construction. Each sample was prepared with 1 ug of total RNA using the Illumina TruSeq mRNA LT Sample Prep kit (Illumina, Inc., San Diego, CA, USA). First, mRNA molecules were purified using poly-T-attached magnetic beads. Then, the mRNA was cut into small fragments with divalent cations. The cleaved RNA pieces were amplified into the first-strand cDNA using SuperScript II reverse transcriptase (Invitrogen, Waltham, MA, USA) and random primers. In the upstream step, the second-strand cDNA synthesis was performed using DNA polymerase I and RNase H. The purified products of PCR reactions were enriched and the final cDNAs libraries were constructed. The RNA-seq libraries were quantified using qPCR according to the qPCR Quantification Protocol Guide (KAPA Library Quantification kits for Illumina Sequencing platforms) and qualified using the TapeStation D1000 ScreenTape (Agilent Technologies, Waldbronn, Germany). Indexed libraries were then sequenced using the NovaSeq6000 platform (Illumina, San Diego, CA, USA). The RNA-seq data have been submitted (https://www.ebi.ac.uk/ena/browser/view/PRJEB80306; accessed on 1 January 2025) to the European Nucleotide Archive under accession no. PRJEB80306.

### 5.3. In Silico Profiling of Urinary Bladder Trigone (UBT) Transcriptome Affected by RTX

High-throughput sequencing was employed for generating paired-end reads. FASTQC v 0.12.1 software was utilized to evaluate raw reads under quality control requirements. Illumina adapters and low-quality reads (PHRED < 20) were eliminated using Trimmomatic v.0.38 [71] according to the following parameters: all sequences were reduced in length to 120 bp, and quality assessments were conducted using 10 bp frame shift increments. Filtered reads were aligned to the reference pig genome by applying ENSEMBL annotation (Sus_scrofa.Sscrofa1.1.1.99) through the STAR program. The alignments of each sample were saved in Binary Alignment/Map (BAM) format. The mapping technique utilized the ENCODE parameters. A minimum 8-nucleotide overhang was necessary for splice junction areas. Introns shorter than 20 bp and longer than 1,000,000 bp were excluded from the analysis. Transcript quantity and structure were subsequently assessed using StringTie v.1.3.3 [72], which involved creating a new GTF file from previously created BAM files and the reference genome. FeatureCounts version 2.0.6 was applied to quantify reads from BAM files.

### 5.4. Differential Expression Profiles and Functional Annotations

The DESeq2 Bioconductor R library was utilized to evaluate the statistical significance of the identified expression in Sus scrofa domestica genes based on previously obtained counts. Genes with adjusted *p*-values less than 0.05 and absolute log2FoldChange more than 1 were deemed statistically significant. The compounds were subsequently categorized based on their coding potential. Molecules were categorized according to gene biotypes from ENSEMBL. A protein-coding gene was categorized as DEG. If the identified RNA transcript was determined to be a long non-coding RNA (lncRNA) and demonstrated statistically significant changes in expression levels between the compared experimental conditions, it was subsequently categorized as DEL. All remaining entities were categorized as other RNA. Co-expression analysis was conducted to ascertain associations between DEGs and DELs and both DEGs and other RNA molecules with analogous transcriptome profiles. A rigorous Pearson correlation coefficient criterion (r > 0.8 and *p* < 0.05) was employed for determining these associated genes.

### 5.5. Differential Alternative Splicing Events Analysis

ASEs were identified using rMATS v.4.1.0 [73]. This software identified and classified A5SS, A3SS, MXE, RI, and SE using a previously created GTF file and BAM files matched to the Sus scrofa domestica reference genome. DASes were characterized by an absolute inclusion level of splicing events (IncLevelDifference) > 0.1 and statistical parameter FDR < 0.05.

### 5.6. Differential Single Nucleotide Variants Analysis

SNVs in transcripts were acquired utilizing the rMATS-DVR v.1.0.0 program (https://github.com/Xinglab/rMATS-DVR; accessed on 1 January 2025) [74]. The study facilitated the assessment of discrepancies between CTR and RTX samples. In the subsequent phase of the bioinformatics study, default parameters were utilized to exclude low-quality SNVs. SNVs situated in the subsequent regions were excluded from the dataset: those within bidirectional genes, 5 base pairs from the splice junction, 50 base pairs upstream and downstream of paralogous sequences. Thereafter, SNVs with AAF > 0 in a minimum of fifty percent of the RNA-seq samples were chosen for additional study. Significant differences in allele expression between the CTR and RTX groups were determined when two criteria were satisfied: |∆AAF| > 0.1 and FDR < 0.05. The Variant Effect Predictor (VEP) was employed to identify SNVs inside the coding sequence region and assess their effects on the transcription of proteins [75].

### 5.7. Functional Annotations

DEGs, DELs, other RNAs, DASes, and ASEs underwent GO functional analysis utilizing the g:Profiler v.0.2.2 software [76]. Processes were deemed statistically significant if the adjusted *p*-value was less than 0.05. GO annotations were designated at three levels: BP, MF, and CC.

### 5.8. Real-Time PCR

The abundance of mRNA transcripts for chosen genes was assessed using Real-Time PCR. We employed the Primer3Plus software 32 (ELIXIR, Hinxton, Cambridgeshire, UK) to design primers for the target transcripts. The primer sequences were based on the information provided in Table S9. Extracted RNA used for transcriptomics analysis was also utilized for validation. cDNA was obtained using the Applied Biosystems™ High-Capacity cDNA Reverse Transcription Kit (Thermo Fisher Scientific, Vilnius, Lithuania, cat. no. 4374966) according to the manufacturer’s protocol. We employed PowerUp™ SYBR™ Green Master Mix (Applied Biosystems™) for real-time PCR (Thermo Fisher Scientific, Vilnius, Lithuania, Cat. No. A25780) according to the manufacturer’s protocol on the QuantStudio™ 3 Real-Time PCR System (Applied Biosystems™, Thermo Fisher Scientific Inc., Waltham, MA, USA). Briefly, each reaction contained 5 μL of master mix (2X), forward and reverse primers at 500 nM of each, 10 ng of cDNA, and an appropriate volume of nuclease-free water to a final volume of 10 μL. Four replicates of each reaction were run on the QuantStudio™ 3 Real-Time PCR System (Applied Biosystems™, Thermo Fisher Scientific Inc., Waltham, MA, USA). Gene expression was quantified with the Pfaffl method [77], and the expression data were presented as the relative change compared to the control group, after normalization to the expression of actin beta (ACTB), used as a reference gene (GenBank accession number U07786.1; relative quantification RQ = 1). Data are shown as mean ± standard deviation on a log10 scale. Statistical significance was determined using a two-tailed Student’s *t*-test, analyzed with Prism 8 software (GraphPad Software Inc., San Diego, CA, USA). *p*-values were considered statistically significant where <0.05 (*), <0.01 (**), <0.0002 (***), and <0.0001 (****). The genes chosen for validation were selected based on their statistical significance in the RNA-seq analysis, biological relevance to RTX-induced molecular changes, and involvement in key pathways identified in gene ontology (GO) and pathway enrichment analyses. Specifically, we prioritized genes associated with nerve function, immune modulation, and synaptic plasticity, as these processes are crucial in understanding RTX’s effects on bladder function.

### 5.9. Sectioning of the Urinary Bladder Trigone Samples and Immunohistochemical Procedure

The tissue samples analyzed in the present experiment were collected from the UBTs taken from the six CTR and six RTX-treated pigs. Ten-micrometer-thick cryostat sections of the samples were air dried at room temperature (RT) for 30 min, postfixed with 4% buffered paraformaldehyde (pH 7.4; 10 min), and processed for single-labeling immunofluorescence (according to an earlier described method [78], using primary antibody against serotonin; Anti-5-HT; dilution 1:10.000; S5545 (Sigma-Aldrich, St. Louis, MO, USA) and Cy™3-conjugated AffiniPure™ F(ab’)2 Fragment Donkey Anti-Rabbit IgG secondary antibody (dilution 1:700; 711-166-152; Jackson ImmunoResearch Laboratories inc.; West Grove, PA, USA)). The slides were coverslipped with Fluoroshield mounting medium containing DAPI (Fluoroshield™ with DAPI, Sigma-Aldrich, St. Louis, MO, USA) for nuclear counterstaining. The labeled sections were viewed under an Olympus BX51 microscope equipped with epifluorescence and an appropriate filter set for CY3. Image acquisition was conducted with an Olympus XM10 digital camera (Tokyo, Japan). CellSens Dimension 1.7 Image Processing software was integrated into the microscope (Olympus Soft Imaging Solutions, Münster, Germany). The distribution and frequency of labeled nerve fibers (NF) were analyzed semi-quantitatively in 10 sections per animal, examining 5 fields within each section. Two independent investigators assessed these structures in the same samples. The number of structures exhibiting immunoreactivity to each substance was evaluated subjectively using a scale from — (when the NF were not found) to ++++ (a very dense meshwork of fibers). When the primary antiserum was omitted or replaced with the corresponding non-immune serum, the specimens did not exhibit any detectable fluorescence.

## Figures and Tables

**Table 1:** The distribution and relative frequency of serotonin-immunoreactive (5-HT-IR) nerve fibers supplying the porcine urinary bladder wall; resiniferatoxin (RTX); +/− single fibers; + few fibers; ++ moderate number of fibers; ↑ an increase in the nerve fibers density.

Part of the Urinary Bladder Trigone	Control Pigs	RTX-Treated Pigs
Muscle layer	+/−	++ ↑
Submucosa	+/−	+ ↑
Urothelium	+/−	+ ↑

## Data Availability

The data underlying this article are available in the European Nucleotide Archive repository, accession number PRJEB80306 (https://www.ebi.ac.uk/ena/browser/view/PRJEB80306; accessed on 1 January 2025).

## Supplementary Materials

The following supporting information can be downloaded at: https://www.mdpi.com/article/10.3390/toxins17030127/s1, Table S1: The summary statistics of cDNA libraries; Table S2: Molecules with statistically significant expression differences provided by RNA-seq; Table S3: Functional annotations of differentially expressed genes DEGs; Table S4: Correlation of differentially expressed genes (DEGs), differentially expressed long non-coding RNAs (DELs), and other RNAs; Table S5: Differential alternative splicing events (DASes) analysis; Table S6: Functional annotations of differential alternative splicing events (DASes); Table S7: Differential analysis of single nucleotide variants (SNVs); Table S8: Functional annotations of single nucleotide variants (SNVs), Table S9: The list of primers used for Real-time PCR.
