# Supplementary material for: Transcriptomic Characterization of the Porcine Urinary Bladder Trigone Following Intravesical Administration of Resiniferatoxin: Insights from High-Throughput Sequencing
Source: Toxins (Basel). 2025 Mar 9;17(3):127. doi: 10.3390/toxins17030127 (PMC11946646; doi:10.3390/toxins17030127)
Supplement: Supplementary file 1 [file toxins-17-00127-s001.zip › Supplemental Table S9.pdf]

Supplemental Table S9. The list of primers used for Real-time PCR.

Name	Forward sequence: (5' to 3')	Reverse sequence: (5' to 3')
FAXDC2	TGAAGGGAGAAGCTGGAAGG	GGTCCACTCCTTCCCTTCAA
INTS7	CGGATTCTGATGTGCCACTG	AGAGTGCTTTCTGACTGGCT
LIMS2	ATGAACAACAACTGGCACCC	CGCTGACAGATGTACTTGCC
MBD5	TCCTGCTGTTGTTCCTTTGC	TCCTTCCAATTCCAGTCCCC
PSEN1	ACGTTGAAATATGGCGCCAA	CAGAATTGAGTGCAGGGCTC
SEPTIN1	CTTGACACAAACGCTGACCA	GCTCGAACTGCTCCTCAATG
